# Dsembler - DNA Assembly Designer: A Tool for Facilitating Assembly of Oligomers

**DOI:** 10.4014/jmb.2412.12046

**Published:** 2025-02-25

**Authors:** Aporva Gupta, Gyeongmin Park, So-Yoon Park, Haneul Kim, Eugene Rha, Dae-Hee Lee, Seung-Goo Lee, Haseong Kim

**Affiliations:** 1 Synthetic Biology and Bioengineering Research Center, Korea Research Institute of Bioscience and Biotechnology (KRIBB), Daejeon 34141, Republic of Korea; 2 Department of Biosystems and Bioengineering, KRIBB School of Biotechnology, University of Science and Technology, Daejeon 34113, Republic of Korea; 3 Department of Chemical Engineering and Applied Chemistry, Chungnam National University, Daejeon, 34134, Republic of Korea; 4 Graduate School of Engineering Biology, Korea Advanced Institute of Science and Technology (KAIST), Daejeon 34141, Republic of Korea

**Keywords:** DNA oligomer assembly, synthetic biology, oligomers, Long-DNA design tool, Dsembler, DNA synthesis

## Abstract

Synthetic biology has garnered significant global interest owing to its diverse applications in bio-based production, biosensing, living therapeutics, and drug delivery. This heightened interest has increased the demand for novel protein synthesis methods and genome-scale assemblies. However, gene assembly from oligomers presents several challenges, including the risk of incorrect assembly between oligomers, self-annealing, and the potential for insertions or deletions resulting from misannealed oligomers. Dsembler (DNA Assembly Designer) is a web-based software application designed to assemble long DNA sequences. It provides a set of oligomers with optimal melting temperatures and GC overlap, which facilitates a commercially available oligomer pool service. By enhancing the accuracy of oligomer design, Dsembler has addressed critical challenges in synthetic biology and supported advancements in genetic engineering and molecular biology.

## Introduction

The primary objective of synthetic biology is to design and construct biological systems that fulfill specific predetermined functions. Progress in this field relies heavily on the ability to assemble short oligomers, which are essential for creating customizable and functional DNA strands. Declining costs of DNA synthesis, along with advancements in computational capabilities, have facilitated rapid developments in the construction of increasingly long DNA sequences. Several DNA assembly techniques such as polymerase chain assembly (PCA), Gibson Assembly, and Golden Gate Assembly have been developed and continue to improve [1]. Although numerous software tools offer the design and analysis of short DNA fragments such as primers [2-5], publicly available software is limited in the number and range of functions. Herein, we present Dsembler (DNA Assembly Designer), a novel software tool that designs optimized oligonucleotide sequences for synthesizing long double-stranded DNA (dsDNA) through polymerase chain assembly (PCA). This tool generates a series of oligonucleotides that can be assembled into a complete gene sequence using PCA methodology. Dsembler generates the appropriate assembly oligomers for a given sequence and provides manual optimization with overlap visualization, allowing users to make more informed decisions. This software is available at http://223.130.146.86:8088/.

## Materials and Methods

### Materials

The PCA and polymerase chain reaction (PCR) were performed using KOD Polymerase (TOYBO, Japan). All enzymatic reactions were performed using a Biometra TRobot II automated PCR cycler (Analytik, Germany).

### Target DNA Sequences and Oligomer Design

The M13 bacteriophage (GenBank Accession No.: NC_003287.2) fragment used to test efficacy was obtained by basic splicing of the M13 bacteriophage genome into 520bp segments. The second fragment, i.e. 521-1041 bp, was used for this study.


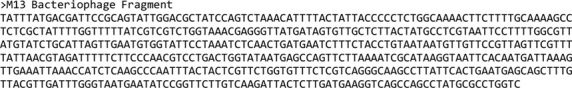


For all design methods, the target oligomer size was 50 bp with an overlap of 20 bp and an overlap Tm of 56°C. While manually designing oligomers, the oligomer that fit the required criteria best was selected. This method is similar to the workflow of Dsembler, however, there were limited candidates to choose from, as designing such sequences was a laborious task. Twenty oligomers were designed by Dsembler, 18 by GeneDesign, and manually for the M13 bacteriophage fragment.

The *aurI* sequence was obtained from *Staphylococcus aureus*. For DNAWorks, the annealing temperature was set to 62°C, and the oligo length was set to 90 nt. For Dsembler, parameters included a maximum oligomer length of 90 nt, maximum overlap length of 40 bp, and Tm for 5'/3' overlap at 58°C.

*aurI* sequences, designed by Dsembler (5' to 3' order):


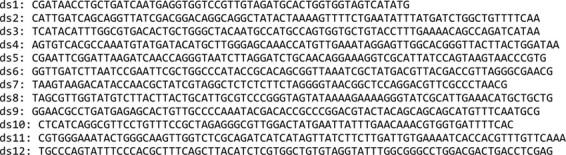


*aurI* sequences, designed by DNAWorks (5' to 3' order):


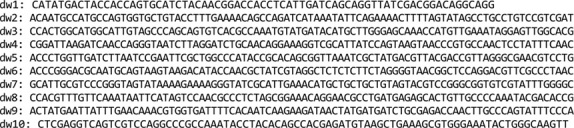


### M13 DNA Assembly

The protocol used to compare the oligomers designed manually, and using GeneDesign and Dsembler were the same, and the assemblies were performed under the same conditions. The synthesized oligomers were pooled depending upon the respective design method. Then, a PCA reaction using Pfu Polymerase (Bioneer, Republic of Korea) was performed on each of the oligomer pools. To amplify the yield and select the correct assembly, PCR was performed (KOD One Polymerase, TOYOBO) using the first and last oligomers as forward and reverse primers. The appropriate sequence was extracted using gel extraction, and the resulting sequences were cloned into a T-vector (Solgent, Republic of Korea) coupled with the lac operon (with kanamycin and ampicillin resistance genes) using Gibson Assembly (NEB, USA). *E. coli* DH5α cells were transformed with the plasmid vector and incubated on an LB agar plate containing 50 ug/ml, 40 ug/ml, 10 ug/ml, 5 ug/ml of IPTG (Duchefa Biochemie, Netherlands), X-gal (Bioneer), ampicillin, and kanamycin (Sigma-Aldrich, USA) respectively, overnight. White colonies, that signified a successful Gibson Assembly, were randomly picked, and underwent a plasmid miniprep (Promega, USA). The final DNA was sequenced using Sanger sequencing, and the obtained sequences were checked for errors.

### aurI DNA Assembly

All experiments were performed using hardware available at KRIBB-biofoundry (Republic of Korea). The workflow involved designing both tools and ordering the oligo pools from Integrated DNA Technologies (USA). Oligos were dissolved in 10 mM Tris-HCl (pH 8.0) and 1 mM EDTA buffer (Bioneer, C-9005, Republic of Korea) to a final concentration of 2 pmol/μl in a total volume of 500 μl using a Janus G3 Liquid handler (Revvity, USA), and they were stored in a 384-well polypropylene plate (Backman, Part No. 001-14615). The PCA mix was aliquoted into a 96-well PCR plate (Bio-Rad, USA), and the oligo pools were transferred using an Echo 525 Liquid Handler (USA) according to their respective final concentrations (2.5, 5, and 25 fmol/μl). The PCA reaction was run at 98°C for 10 s, then 19 cycles of 98°C for 10 s, 55°C for 30 s (ramp rate: 0.2°C/s), and 68°C for 30 s, followed by a single incubation at 68°C for 3 min. Primers aligned to the ends of the DNA assemblies were used to amplify the PCA products. Then, 2 μl of each product was run through a fragment analyzer (5400 Fragment Analyzer System, Agilent) with a dsDNA 920 Reagent Kit (75–15,000 bp) to observe the size and concentration of the final assembly.


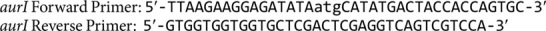


**Abbreviations:** dsDNA, double-stranded DNA; Tm, melting temperature; PCA, polymerase chain assembly; PCR, polymerase chain reaction

## Results and Discussion

### Designing Optimized Oligomers

**Fig. 1:**
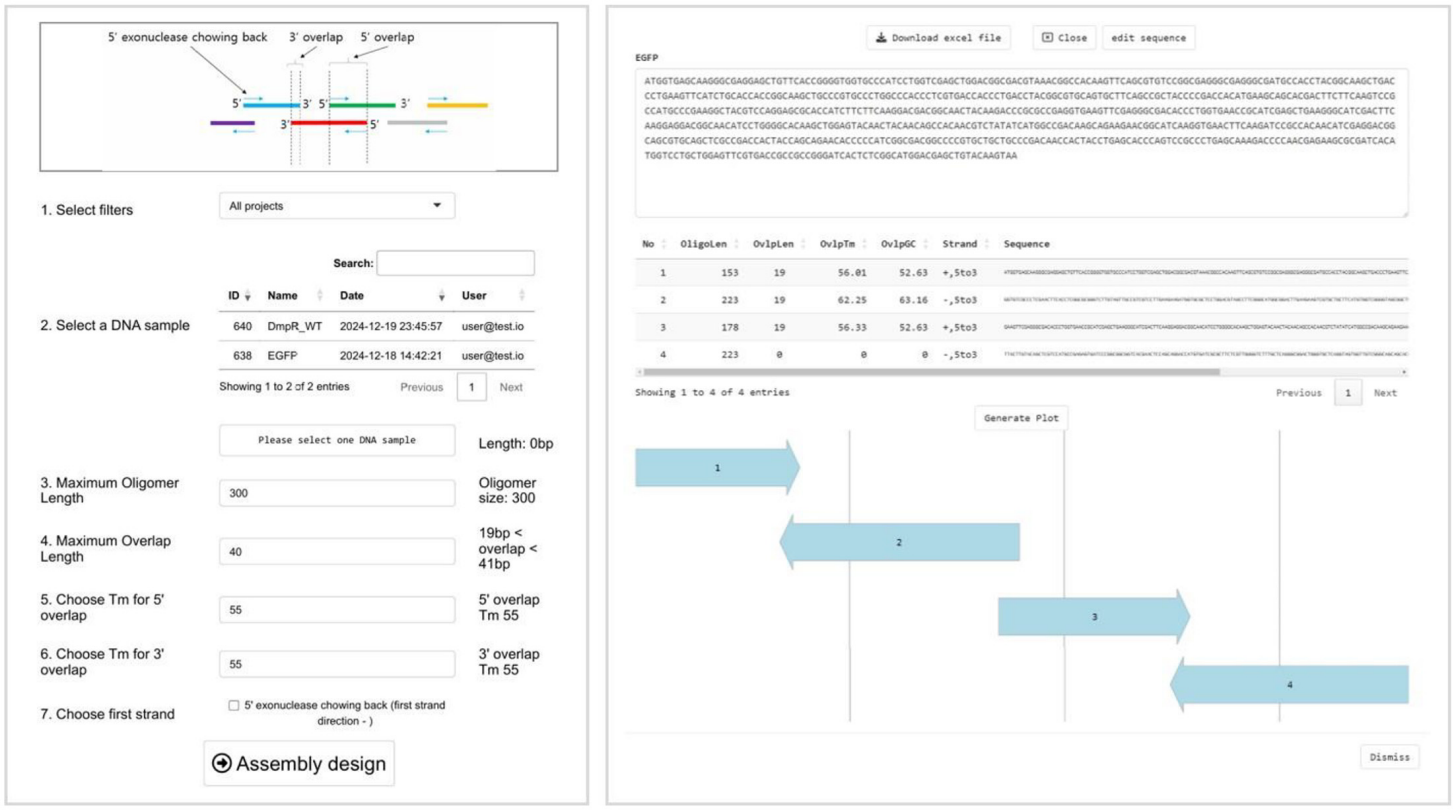
Dsembler user interface for assembly design and result display.

Dsembler was developed using R (version 4.1.2) with the lightweight Shiny web user interface and MongoDB for database storage using the Mongolite package (Fig. 1). Users can create a project and upload FASTA format sequence samples within the project. A set of oligomers can be designed by providing inputs for the target DNA sequence, oligomer size, overlap size, and melting temperature (Tm) range. Users can manually optimize Tm and GC content overlap using a visualized sequence. Users can register on Dsembler to effectively store, manage, and analyze their queries.

The main considerations of Dsembler for optimizing oligomers include the following: 1) Designing oligomers while avoiding possible errors in DNA assembly: The central idea of the algorithm is to generate an array of all possible candidate oligomers within the given parameters and select those that best fit the parameters. Ideally, oligomers should not contain thymine as the last nucleotide of the 3' end, have an overlap GC content between 40% and 60%, and a Tm within the range specified by the user. If no oligomers are suitable, the next-best fit is provided. Tm is calculated based on the Nearest Neighbor Equation supported by the SantaLucia6 thermodynamic table [7]. 2) Preventing the false assembly of oligomers: Dsembler prevents the false assembly of oligomers that share complementary base pairs between their overlaps. Consecutive oligomers are grouped and segregated based on the presence of repeat sequences (10 bp) between the oligomer overlaps in each group. 3) Penalty score for assessing assembly errors: A penalty score for assessing potential assembly errors is provided in advance. The scores for each oligomer are calculated using a weighted sum of the possible causes of errors that may occur during assembly. Scoring is performed using the following equation:

$$score=\left|T_{t}-T_{c}\right|+1_{T}+GC_{clamp}+R_{within}+R_{between},$$ (1)

where *T_t_* and *T_c_* are the target overlap Tm and calculated overlap Tm, respectively, and 1*_T_* represents an indicator function, which is 1 if the base at the 3' end of the oligomer is thymine; otherwise, it is 0. *GC_clamp_* refers to the number of GC clamps in an overlapping region. *R_within_* represents the number of repeat base pairs in each oligomer. *R_between_* represents the number of repeat base pairs between the overlaps in a cluster. A theoretically faultless oligomer that fits all the target parameters will have a score of 0.

### Dsembler Interface

Users can register on Dsembler to store, manage, and analyze their queries effectively. The "Project" tab enables users to manage queries on a project-specific basis. The "Sample" tab allows for the upload of DNA sample files in FASTA format or directly via a dialog box. Within the "Design" tab, users can design oligomers by specifying preferred conditions, including melting temperature (Tm), maximum oligomer length, and maximum overlap length for the uploaded samples. The "Assembly" tab offers a visualization of the assembled DNA data through tables and graphs, with the option to download results in Excel format. Possible errors and the resulting quality score for each oligomer is also provided in this section. Finally, the "Optimizer" tab allows users to manually optimize oligomers identified as error-prone. This would be particularly useful when constructing oligomers for more complex sequences such as high GC-content sequences or repetitive sequences. The resulting oligomers are presented as 5' to 3' sequences in CSV format, facilitating the ordering of oligomer pool samples.

### Experimental Evaluation

A comparative study was performed among manually designed oligomers, GeneDesign3, and Dsembler. Oligomers of a predefined 520 bp fragment of the M13 bacteriophage genome, designed using the aforementioned approaches, were synthesized and assembled using PCA. The expected oligomer size, overlap size, and overlap melting temperature were set to 50 bp, 20 bp, and 56°C, respectively. The three design methods were assessed by checking their insertion and deletion rates (errors/kb) after sequencing the assembled DNA obtained from the colonies. The proportion of randomly sampled colonies with no errors was also checked (Table 1). For this sequence assembly, Dsembler was seen to be relatively successful with no insertions recorded and minimal deletions (0.38 ± 0.5 errors/kbp). Dsembler oligomers were observed to have a notably lower error rate compared to GeneDesign (2.02 ± 1.0 errors/kbp) and manually designed oligomers (0.67 ± 0.6 errors/kbp). Although the reduced error rate correlates to an improved design, it is yet unclear if the errors are caused due to the oligomer design or subsequent PCR step to amplify the assembled sequences. Such single base mismatches can be improved with an enzyme treatment such as T7 endonuclease. The proportion of error-free colonies from the Dsembler oligomers (83%) was comparable to that of the manually designed oligomers (71%), and more than double of GeneDesign oligomers (33%). A high proportion of error free colonies imply fewer experimental steps during assembly, particularly for error corrections. This would accelerate long DNA assembly and be useful for high throughput protocols.

**Fig. 2:**
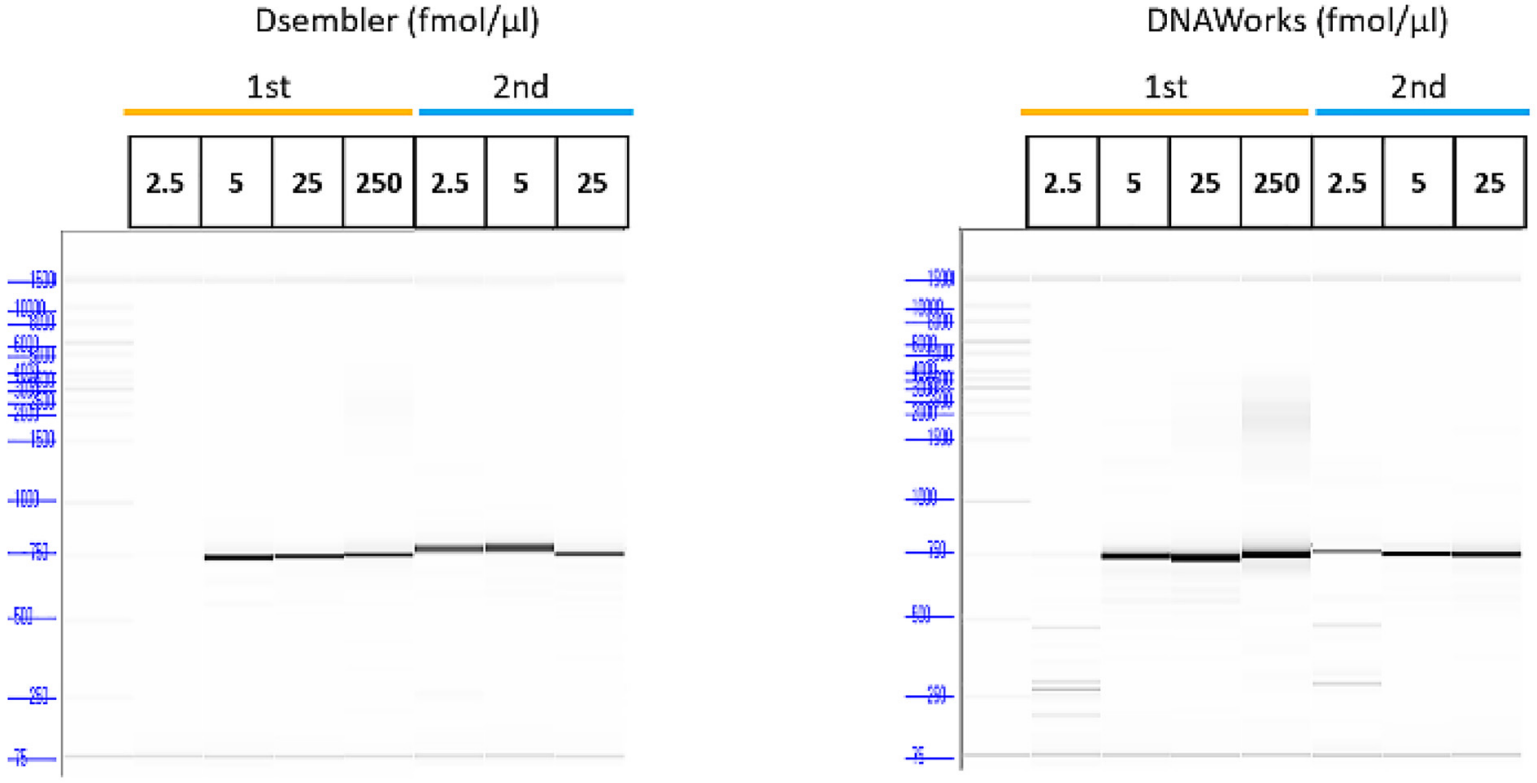
Fragment analysis results for the Dsembler and DNAWorks designs. The electropherogram traces show the DNA bands of the PCA-based assembled DNA products from Dsembler and DNAWorks. The y-axis refers to the size of the DNA. Each tool’s oligomers were assembled with varying concentrations of 2.5, 5, 25, 250 fmol/μl over 2 separate trials.

The established DNAWorks tool was compared with Dsembler within the KRIBB-biofoundry. Both tools were used to design the *aurI* gene (699 bp) fragments (12 and 10 oligomer fragments from Dsembler and DNAWorks, respectively). The designed gene fragments were synthesized and then pooled and assembled using PCA (see the details in the Methods section). The assembled gene fragments were analyzed using a fragment analyzer to assess whether the assembly was successful (Fig. 2). We confirmed that oligomers from both Dsembler and DNAWorks were successfully assembled.

### Summary

Oligomer design is a crucial step in increasing the efficiency of long DNA assemblies. Our DNA Assembly Designer, Dsembler, utilizes a robust algorithmic design that effectively generates optimal oligomers for DNA assemblies. It features an easy-to-navigate user interface with default values to guide users in each step. Oligomer visualization allows for a clearer understanding of how the assembly will occur. By integrating it with automated machinery in a biofoundry, we can scale up assembly outputs as well. Future work will consider the formation of secondary structures, such as hairpins, loops, and other intramolecular interactions, which can interfere with the correct binding of oligomers or the intended assembly order. Secondary structures can lead to incomplete or erroneous assemblies by hindering polymerase activity or causing mispriming during synthesis. This could involve integrating existing secondary structure prediction tools, such as Palindrome analyzer [8], or incorporating real-time structure analysis into the assembly design process to ensure that potential structural obstacles are accounted for and minimized.

## Figures and Tables

**Table 1:** Oligomer design analysis.

Design method	Insertion rate	Deletion rate	Total error rate	Error free colonies (%)
Dsembler	0	0.38 ± 0.5	0.38 ± 0.5	83
GeneDesign	1.13 ± 0.7	0.9 ± 0.7	2.02 ± 1.0	33
Manual	0.40 ± 0.6	0.27 ± 0.4	0.67 ± 0.6	71

**Table 2:** Comparison with existing tools.

Design method	Accessibility	Oligomer designing time (min)	Visualization	Batch processing
Dsembler	Webserver	Negligible	Available	Yes
GeneDesign	Webserver (currently inaccessible)	Negligible	Not available	Yes (currently inaccessible)
DNAWorks	Terminal access	Negligible	Not Available	Yes
Manual	Requires DNA visualization tools	5 * n	-	No
